# A radioanatomical study of 3rd segment terminal branches of the maxillary artery in the pterygopalatine fossa

**DOI:** 10.1038/s41598-023-29975-1

**Published:** 2023-02-28

**Authors:** Kolos Lovász, Péter Magyar, Tibor Szalóki, Pal Maurovich-Horvat, Károly Altdorfer, László Tamás, Alán Alpár

**Affiliations:** 1 Department of Anatomy, Histology and Embryology, Semmelweis University, Budapest, Hungary; 2 Department of Medical Imaging, Semmelweis University, Budapest, Hungary; 3 Department of Otorhinolaryngology and Head and Neck Surgery, Semmelweis University, Budapest, Hungary

**Keywords:** Dentistry, Medical imaging, Anatomy, Musculoskeletal system, Oral anatomy, Risk factors

## Abstract

This study describes the clinical anatomical topography and relationship of the terminal branches of the maxillary artery to the bony wall of the maxillary sinus in the pterygopalatine fossa (PPF) to estimate the bleeding risk during surgical interventions. Using contrasted computer tomography records, (i) the route of the maxillary artery in the infratemporal fossa, (ii) the number of the arteries in the critical PPF surgery plane, (iii) the diameter of the largest artery in the area and (iv) its relation to the posterior wall of the maxillary sinus were examined. Furthermore, measurements were extended with (v) the minerality of the bony posterior wall of the maxillary sinus on bone-window images. For statistical analyses Student’s t- and Fisher-test were applied. 50 patients (*n* = 50, 100 cases including both sides) were examined in this study. The maxillary artery reached the pterygomaxillary fissure on the lateral side of the lateral pterygoid muscle in 56% of the cases (*n* = 32), in 37% (*n* = 23) on its medial side and in 7% (*n* = 4) on both sides. The number of arteries at the level of the Vidian canal in the PPF varied between 1 and 4 with a median of 2. The diameter of the biggest branch was 1.2–4.7 mm, the median diameter was 1.90 mm. In 41% (*n* = 30) of the cases the biggest artery directly contacted the posterior wall of the maxillary sinus, and the mineral density of the posterior wall was decreased in 14.3% (*n* = 12) of all investigated cases. The present description and statistical analysis of the vasculature of the PPF optimizes operative planning—like clip size or the type and direction of the surgical approach—in this hidden and deep head/neck region.

## Introduction

The pterygopalatine fossa (PPF) is an inverted pyramid-shaped bony space located in the deep face between the maxilla, palatine bone and the pterygoid process of the sphenoid bone. Vessels and nerves enter this space from both the extra- and the intracranial space, which pass further towards nasal and oral cavities, as well as into the orbit^1^. The exact anatomical borders and connections of this hidden area are demonstrated in Table 1^1^.

**Table 1:** Main borders and openings of the pterygopalatine fossa^1^.

	Border	Opening	Direction
Anterior	Posterior wall of the maxillary sinus	Inferior orbital fissure	Orbit
Superior	Greater wing of the sphenoid bone	Foramen rotundum	Middle cranial fossa
Medial	Perpendicular lamina of the palatine bone	Sphenopalatine foramen	Nasal cavity
Lateral	Open	Pterygomaxillary fissure	Infratemporal fossa
Posterior	Greater wing and the pterygoid process of the sphenoid bone	Pterygoid (Vidian) canal, Palatovaginal canal	External skull base
Inferior	Open	Greater and lesser palatine canals	Oral cavity

Radiological and surgical perspectives urged physicians to further subdivide the narrow PPF. To facilitate exact spatial localization, Chen et al. divided the PPF into four portions in the axial plane^2^. Roberti et al., in turn, distinguished an anterior vascular from a posterior neural space^3^. The main element of the posterior space is the sphenopalatine (or pterygopalatine) ganglion which is critical in the innervation of the lacrimal, palatine and nasal glands. The postganglionic parasympathetic fibres are distributed along the branches of the maxillary nerve, which branch off from their stem in the PPF. The anterior vascular division contains the terminal branch of the maxillary artery which, after entering the PPF, splits into its end branches: (a) the posterior superior alveolar artery, infraorbital artery, and artery of the Vidian canal; (b) the descending pharyngeal artery; (c) the sphenopalatine artery and the greater and lesser palatine arteries. The descending pharyngeal artery and the palatovaginal artery can derive from any segment. Of note, the remaining space of the PPF is filled with the rich venous pterygoid plexus, as well as with lymph vessels and fat tissue.

Due to its connections between numerous clinically important spaces, PPF has a key role in the spread of various (inflammatory or tumorous) diseases. Clinically, PPF represents an important area where patients commonly develop a broad of benign and malignant lesions. Most frequently, juvenile angiofibromas develop in this region which, despite their benign nature, show high expansion rate and high vascularization^4^. Juvenile angiofibromas typically affect Asian males with a 0.05% prevalence among all head-and-neck neoplasms^5^. Albeit less frequently, schwannomas, meningiomas, neurofibromas, osteosarcomas and other skull base neoplasms can also develop or spread in the PPF, which mostly occur in elderlies of both sexes^6^. In addition to tumours, the sphenopalatine ganglion has been implicated in different types of headaches and appears as a promising target in these conditions^7^.

Based on CT image analysis, this study aimed to give a clinical anatomical description of the vasculature of the PPF to help surgical intervention.

## Objects

The relationship between the branches of the maxillary artery and the posterior wall of the maxillary sinus, including mineralization was examined. Their vascular trauma decreases intraoperative visualization and causes blood loss during surgery. Based on the present data analysis, an estimation to proper clip size is given, for safe and efficient branch ligature.

## Materials and methods

**Figure 1:**
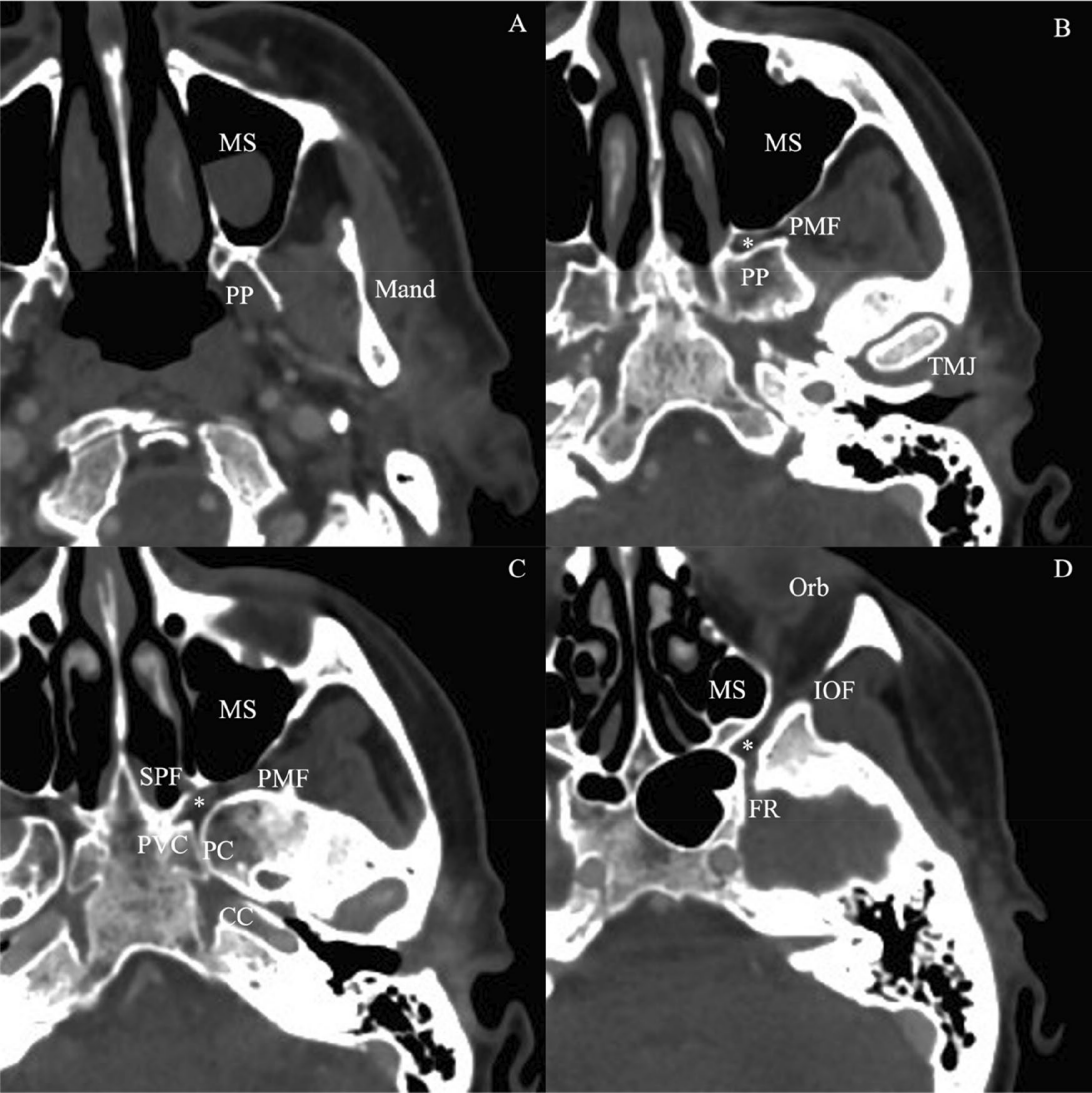
Radiologic anatomy of the pterygopalatine fossa at different levels on axial CT records. (**A**) Immediately above the hard palate, (**B**) at the level of the temporomandibular joint, (**C**) at the level of the carotid canal (petrous segment), (**D**) at the level of the orbit. (WW 1300 ± 80 HU; WL 500 ± 80 HU). *CC* carotid canal, *FR* foramen rotundum, *HU* Hounsfield unit, *IOF* inferior orbital fissure, *Mand* mandibula, *MS* maxillary sinus, *Orb* orbit, *PC* pterygoid canal, *PMF* pterygomaxillary fissure, *PP* pterygoid process, *PVC* palatovaginal canal, *TMJ* temporomandibular joint; *pterygopalatine fossa.

This study is based on retrospective data analysis conducted in October 2021. The studied population consisted of 50 adult patients undergoing acute head-and-neck computed tomography- (CT) examination for neurovascular imaging (e.g., to exclude stroke events) Fig. 1. Exclusion criteria included masses of any origin in the head-and-neck region to support measurements among normal anatomical circumstances. Data were collected from the electronical records of Medical Imaging Centre, Faculty of Medicine, Semmelweis University, Budapest, Hungary. Patients were examined between June 6th and September 18th, 2021.

Following parameters were collected: number of visible arteries in the level of the Vidian canal; diameter of the biggest identified artery; and the relationship of the artery to the posterior wall of the maxillary sinus. In addition, it was also explored and described how the maxillary artery reached the pterygopalatine fossa. In a subgroup analysis (42 patients, i.e., 84 sides), the mineralization of the posterior wall of the maxillary sinus was also evaluated. Possible values and the number of investigated records are listed in Table 2.

**Table 2:** Study characteristics.

Examined parameter	Possible values	Number of investigated images (with respective patient numbers)
Route of the maxillary artery between the medial side of the mandibula and the pterygomaxillary fissure	Medial/lateral/both side(s) of the lateral pterygoid muscle	100 (n = 50)
Number of arteries at the level of the Vidian canal	Quantity	100 (n = 50)
Diameter of the greatest artery	Expressed in millimetres (mm)	100 (n = 50)
Relationship between the biggest artery and the posterior wall of the maxillary sinus	Direct/indirect relationship	100 (n = 50)
Mineralization of the posterior wall	Decreased/not decreased in comparison to its surroundings	84 (n = 42)

The selected patient group consisted of 30 males and 20 females with a median age of 72 years and with an interquartile range of 13.5 for men and 19.75 for women. No further data was collected beyond above listed specifics.

### Radiological methods

50 contrast enhanced head-and-neck CT images were used, made on Philips Incisive CT128 and Philips Brilliance CT64. Contrast enhanced records were expanded with the corresponding native and ‘bone window’ images. Measurements were performed on a DICOM image pool, using the default tools in IMPAX software. Several measurements were documented also with screenshots. Radiologic specifics are shown in Table 3.

**Table 3:** CT specifications regarding contrast-enhanced and bone-windowed records.

	Contrast-enhanced records	Bone-windowed records
Slice thickness (mm)	1	1
Space between slides (mm)	0.85	1
Spacial resolution (mm)	0.33	0.33
Pixel spacing (mm)	0.40/0.40	0.41/0.41
Convolution kernel	UB	YB

### Statistical analyses

After extracting raw data, several statistical analyses were performed. Since age and vessel diameter as biological parameters showed normal distribution, we performed Student’s t-test. With qualitative parameters we ran a Fisher’s test. These parameters were the route of the maxillary artery, number of blood vessels in the PPF, topographical relation and minerality. Results were considered significantly different at a level of *p* < 0.05.

### Ethical statement

This study is based on retrospective data analysis. Images were captured during routine clinical investigations and patients remained anonymous in this work. All investigations were executed according to the Ethical Guideline of Semmelweis University and authorized by the Regional and Institutional Committee of Science and Ethics (SE RKEB number 180/2022). The Regional and Institutional Committee of Science and Ethics approved our request and the informed consent was waived.

## Results

Images of a total of 50 patients were collected between June 2021 and September 2021 (Fig. 1). The selected patient group consisted of males (60%, *n* = 30) and females; the median age was 72 years with an interquartile range (IQR) of 16.

No mass of any origin in the head-and-neck region was found in the 50 records. In a single case, the nasopharyngeal tube was visible, which, however, left all anatomical landmarks in the PPF and its region unaltered.

### The maxillary artery in the infratemporal fossa

**Figure 2:**
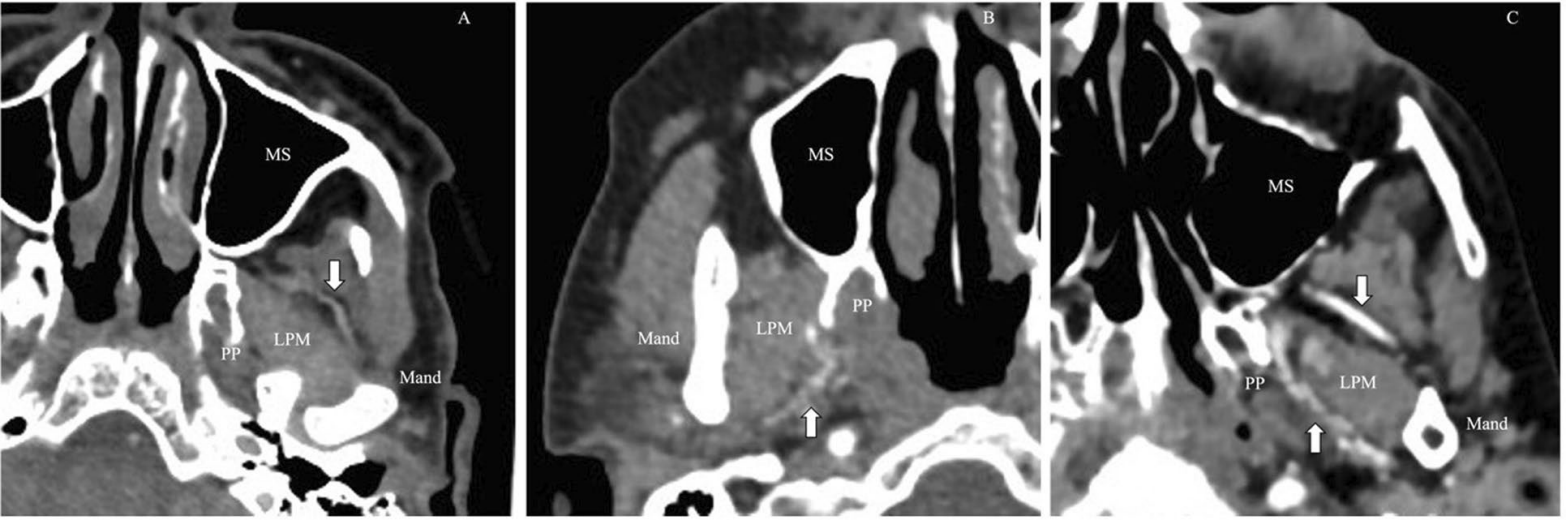
Route of the maxillary artery in the infratemporal fossa. (**A**) only lateral (left side), (**B**) only medial (right side), (**C**) on both sides (left side). (WW 1300 ± 80 HU; WL 500 ± 80 HU) Abbreviations and symbols: *HU* Hounsfield unit, *LPM* lateral pterygoid muscle, *Mand* mandibula, *MS* maxillary sinus, *PP* pterygoid process; arrow: the examined artery.

The maxillary artery was visible constantly on the medial side of the mandibula. In the first variation, it coursed along the lateral side of the lateral pterygoid muscle and then turned medial immediately at the pterygomaxillary fissure to enter the PPF. In the second variation, the artery passed on the medial side of the lateral pterygoid muscle, turned, and passed lateral to reach the pterygomaxillary fissure before entering the PPF. Both variations occurred simultaneously, when arteries passed on both sides of the lateral pterygoid muscle. These variations are seen on Fig. 2.

On the 50 records (100 PPF, both sides in total), 56 (*n* = 32) arteries reached the pterygomaxillary fissure on the lateral and 37 (*n* = 23) arteries on the medial side of the lateral pterygoid muscle. In 7 (*n* = 4) cases a bifurcating maxillary artery could be observed. This means, the maxillary artery coursed on both sides on the lateral and medial aspects of the lateral pterygoid muscle in 24 (48%) and 14 (28%) patients, respectively, whereas in 3 patients (6%) a bifurcating variation could be identified on both sides. Finally, in 9 patients (18%) a different distribution pattern was found between the right and left sides. No statistically significant differences were confirmed (*p* = 0.59, Fischer’s test).

### Number and diameter of arteries in the PPF

For the rest of the measurements, the level of the Vidian canal was used. The maxillary artery, after its meandrous path, enters the PPF to divide finally into its 3rd segment terminal branches. Although the number of arteries is not a certain indicator for the branching pattern, it likely correlates with the extent of a potential bleeding.

At the critical level, the median number of arteries was 2 (with a range between 1 and 4). In 39 cases (*n* = 26) a single artery was observed, in 30 cases (*n* = 24) 2 branches appeared, whilst 3 and 4 branches were identified in 24 (*n* = 18) and 7 (*n* = 5) cases, respectively. The number of the arteries did not differ either between sides [2.0 (2) on the right side vs. 2.0 (2) on the left side, median (IQR)] or between sexes [2.0 (2) in male vs. 2.0 (2) in female, median (IQR)]. Fisher’s test with Freeman-Halton extension using a 2 × 4 contingency table explored no significant differences (*p* = 1 and *p* = 0.74 for side and sex comparisons, respectively).

The diameter of the arteries was also measured in order to choose the largest one (as most likely responsible for a significant bleeding) for further examinations. The median value was 2.05 mm with a range between 1.2 and 4.7 mm, without significant side differences [1.90 (0.56) mm on the left side vs. 1.90 (0.80) mm on the right side, median (IQR)]. The diameter of the largest artery was similar in both sexes [2.05 (0.58) mm in male vs. 1.8 (0.45) mm in female, median (IQR)]. No significant difference was explored in either relation (*p* = 0.87 and *p* = 0.55 for side and sex comparisons, respectively).

### The relationship between the posterior wall of the maxillary sinus and the largest artery

**Figure 3:**
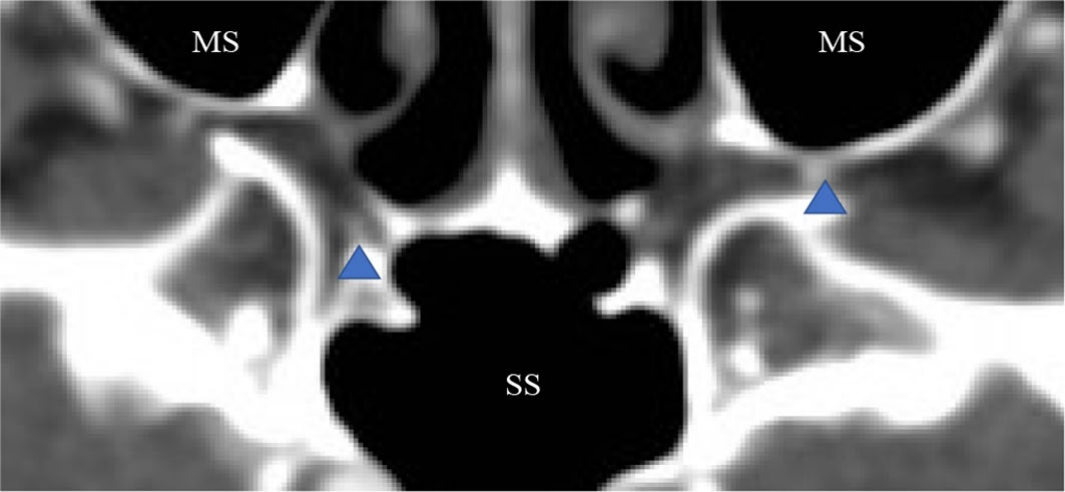
Relationship between the largest artery and the posterior wall of the maxillary sinus. (WW 1300 ± 80 HU; WL 500 ± 80 HU). *HU* Hounsfield unit, *MS* maxillary sinus, *SS* sphenoidal sinus; arrowhead: the examined artery.

Parameters of the largest artery were further examined. To avoid ambiguity when estimating its relationship to the maxillary sinus the following methodology was applied: the lack of any sign of soft tissue between the examined artery and the posterior wall of the maxillary sinus was documented as “direct relation”, other cases were identified as “indirect relation” (see also in Fig. 3).

Amongst the total of 100 investigated images, 41 vs. 59 direct and indirect cases, respectively, were identified. Thus, in 41% of the cases (*n* = 11), the artery directly contacted the bony wall of the maxillary sinus without any further soft tissue in between.

In 11 from 50 patients (22%) the largest artery had a direct relation to the posterior wall of the maxillary sinus on both sides, while in another 20 (40%) patients there was an indirect relationship on both sides. In the last group of 19 (38%) patients the biggest measured artery had a larger diameter on the left (13 of the 19, 68.4%) versus the right side. No significant differences were found in terms of direct or indirect relation of the artery to the posterior wall (Fischer’s test, *p* = 1).

### Mineralization of the posterior wall of the maxillary sinus

**Figure 4:**
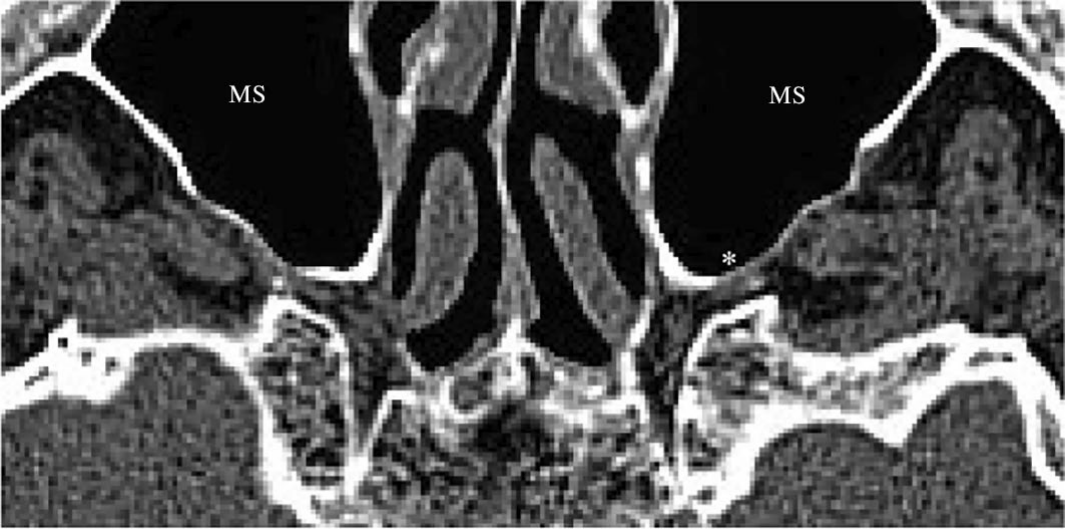
Minerality of the maxillary sinus (WW 1000 ± 80 HU; WL 300 ± 80 HU). *HU* Hounsfield unit, *MS* maxillary sinus, *decrease in bone density on the left posterior wall of the maxillary sinus.

The mineralization of the posterior wall of the maxillary sinus was explored at identical craniocaudal level with the previous set of measurements (Fig. 4). The radiation intensity of the posterior wall of the maxillary sinus was compared with the radiation intensity of maxillary bone tissue unrelated to the PPF (for this purpose, the lateral wall of the maxillary sinus was used).

Bone-windowed records were missing in 8 patients, which means, minerality was measurable on only 84 sides. In this subgroup analysis, 24 men and 18 women (a total of 42 patients) were included. The median age of patients was 73 years with an interquartile range of 14. Although the minerality of the bony posterior maxillary sinus wall did not change in 72 (85.7%) cases, minerality decrease occurred in 12 cases (14.3%), with 4 cases being on the right side and 8 cases on the left side. Men and women were affected similarly [6 (7.1%) cases in both sexes], with decreased minerality occurring predominantly on the left side [4 (4.8%) cases in both sexes] compared to the right side [2 (2.4%) cases in both sexes].

Minerality decrease in the posterior wall of the maxillary sinus appeared unilaterally in 8 patients (19%), bilaterally only in 2 patients (4.8%), but was absent on both sides in 32 patients (76.2%). Finally, side difference in minerality decrease emerged in 4 male and 4 female patients, predominantly on the left side (in 6 cases in total). Most critically, the largest artery directly contacted the bony posterior wall of the maxillary sinus in 5 patients with decreased minerality at this locus. Overall, no statistical significance was shown (Fischer’s test, *p* = 1 for both sex and side comparisons).

## Discussion

This study evaluated the vasculature in the PPF of 50 patients. The number and maximal size of the arteries—measured identically at the level of the Vidian canal—varied between 1 and 4 with a median of 2, and between 1.2 and 4.7 mm with a median diameter of 1.90 mm, respectively. These values were rather constant with negligible statistical differences between sides and sexes. Largest sized arteries directly contacted the posterior wall of the maxillary sinus 41% (*n* = 30) of the cases. Further, the mineral density of the bony tissue decreased in 14.3% (*n* = 12) of the investigated cases at this location.

Contrast enhanced CT records are typically used for neurovascular imaging. Nevertheless, this method also allows to visualize branches of the external carotid artery, i.e., vessels with bony or soft tissue surroundings. Tracing the route of the maxillary artery in the infratemporal fossa not only allows better understanding of anatomical relations in the deep face, but critically supports transcranial and infratemporal fossa surgical approaches. The situation with the artery coursing along the lateral aspect of the lateral pterygoid muscle (as observed in 63% of all cases) means a surgically more vulnerable state compared with cases when the artery passes on the medial aspect of the same muscle. Although previous studies mentioned the above anatomical variation^8^, no former description of a bifurcating variation of the maxillary artery was found.

Measurements were carried at a particular level of the PPF, i.e., at the orifice of three canals: the Vidian canal, the palatovaginal canal and the sphenopalatine foramen. In this plane the sphenopalatine ganglion can be depicted anterior to the Vidian canal^9^. Thus, this level is crucial in intraoperative visualization for endoscopic anterior surgical approaches^10^ as well as diagnose select headache types^10,11^. Of note, vegetative/cranial parasympathetic function loss due to intraoperative nerve injury develops as a typical postoperative complication^12^.

The PPF contains many terminal arteries^13^. Although axial image analysis alone may not allow complete reconstruction of different branching patterns, these observations might add a useful and new aspect to surgery planning. That is, bleeding risk manifested in suboptimal visualization and increased blood loss depends on the number of arterial branches in the operative field. In addition to the important information about the number of identifiable major vessels in the pterygopalatine fossa, here it is shown that the bleeding risk (irrespective of any other factors with an influence on haemostasis) depends neither on gender nor on the side of the operation.

The artery with the largest diameter is the best candidate when it comes to intraoperative ligation or to treat posterior epistaxis. Application of proper clip size is essential both to effectively reduce bleeding and to preserve neighbouring structures. Our findings show that the diameter of largest artery shows negligible variation in the area with a mean diameter of 2.0 mm, which allows a firm prediction when choosing appropriate clip size for intraoperative ligation. This should be performed carefully, regarding the unpredictable course of the maxillary artery in the PPF. In addition, as the findings of Isaacs et al. enlighten, the mean distance of the maxillary artery from the sphenopalatine foramen was only 10 mm^14^.

The relation between the posterior wall of the maxillary sinus and especially the biggest artery forecasts the risk of vascular damage during a transantral approach. Previous studies reported that the maxillary artery courses immediately behind the posterior wall of the MS^12,14,15^ but failed to report about the surgically critical actual contact of these terminal branches. Although our data suggest that no such risk applies in more than half of all cases, there is a 41% chance that the largest artery has a direct connection with the posterior wall of the maxillary sinus at least on one side, with higher probability on the left side.

According to the examination on the minerality of the maxilla, any decrease occurs in only 14.3% of all cases, predominantly on the left side. Decreased minerality forecasts increased risk during the surgical approach due to increased vulnerability and less protected vessels. Several authors are concerned about the dehiscence of the surrounding bony structures, e.g., when operating in the region of the sphenoid sinus^16,17^ or at the orbital surface of the maxilla^18^. It is suggested that the minerality of the posterior wall of the maxillary sinus carries direct relevance for a potential surgical intervention in closeness to the PPF. Decreased minerality in the maxilla coupled with large arterial diameter suggest an especially high risk during surgical approach which could be identified in five subjects in our analysis.

One of the limitations of this study is that measurements were performed in a single country (Hungary) with a relatively homogenous patient group, hence, no differences by race was examined which could contribute to anatomical variation etiology. A further limitation may arise from the number of patients involved in the analysis. Statistical tests carried out on larger sample size may reveal minor, subtle differences which could have remained unexplored in this study.

Despite these limitations, this study shows several strengths. A new methodology was applied to objectify the relationship between a vessel and a bony surface on contrast-enhanced CT images. This allowed to recognize that decrease in the mineralization of the bony wall carries new information about bony dehiscence of the skull base. Finally, a third anatomical variant regarding the course of the maxillary artery in the infratemporal fossa is reported for the first time.

## Summary

PPF is a hidden area in the deep face at the base of the skull. Based on high resolution CT imaging, a detailed clinicoanatomical description of the vasculature is given, at a surgically critical plane of the PPF. In addition to the number of vessels at this locus, it is enlightened that large calibre arteries can intimately approach the wall of the maxillary sinus which can parallel decreased mineralization. The present anatomical description, paired with sex- and side specific statistical analysis, may help surgical intervention and subsequent therapy.

## Data Availability

The datasets used and/or analysed during the current study available from the corresponding author on reasonable request.
